# The Impact of Darwinian Evolution on Medicine: The Maternal Side of the Story

**DOI:** 10.5041/RMMJ.10010

**Published:** 2010-07-02

**Authors:** Dan Mishmar

**Affiliations:** Department of Life Sciences and the National Institute of Biotechnology (NIBN), Ben-Gurion University of the Negev, Beer Sheva, Israel

**Keywords:** evolutionary medicine, mitochondria, next-generation sequencing, disease, evolution

## Abstract

Complex disorders are common in the human population and are caused by interplay between genetic and environmental factors. Therefore the quest for the genetic basis of such disorders has much similarity to deciphering the genetic basis of macro-evolutionary processes, such as speciation. Here I discuss conceptual connections between the principles underlying and processes occurring in disease and evolution. Special focus is given to the tremendous mitochondrial genetic variability in the population and within individuals and the impact of both types of variability on evolutionary processes and diseases.

In his *Origins of Species* Charles R. Darwin did not use the term ‘evolution’. During the nineteenth century, when the book was published, this term was mostly used referring to embryo development, and Darwin made every effort to avoid this connection. Instead he focused on persuading the reader that the emergence of new species should be governed by inheritance of changes, the establishment of (inherited) variation, and response to natural selection. Following elucidation of the principles of modern genetics, Darwin’s concepts assimilated into the field of population genetics. Accordingly, the formation of natural genetic variation by mutations and their dispersal by migrations and eventual genetic drift are fundamentally essential to the ability of any given species to cope with environmental changes and selective pressures. Genetic variation increases the odds to form genotypes that would successfully survive environmental shifts. The same logic applies to the emergence of diseases: inspection of the commonly used database Online Mendelian Inheritance in Man (OMIM – www.ncbi.nlm.nih.gov/omim/) reveals that functional alterations of gene products causing Mendelian-inherited diseases could result from multiple independent mutational events thus creating a repertoire of disease-causing allelic variants. The interplay of these disease-causing alleles with the environment and other genetic factors frequently leads to phenotype variability or change in disease penetrance. Complex disorders, on the other hand, could be caused by combinations of multiple mutations in the genetic material in multiple loci, of which some are inherited and some accumulate during the lifetime of the individual. Since a subset of the genetic variations that accumulate during the course of time carries functional properties similar to disease-causing mutations,^1^ and either type of genetic alterations interacts with the environment, it is possible that similar principles govern the emergence and evolutionary survival of disease-causing mutations and genetic variants (Figure 1).

We recently underlined features common to the formation of disease-causing mutations and genetic changes that drive speciation events,^2^,^3^ and therefore I shall only touch upon these issues in the current essay. Instead, I will argue that principles and ways of thinking learned during the last 150 years since the emergence of the theory of evolution should be utilized in modern medicine. Moreover I will show (below) that not only the principles but, even, the same genetic changes could play a role both in disease and evolutionary processes.

## THE DISCIPLINES OF MODERN MEDICINE AND THE CONFUSION WHILE DESIGNING THE TREATMENT FOR COMPLEX DISEASES

Modern medicine and biomedical research have emerged during the eighteenth–nineteenth centuries when the first vaccines were developed. Specifically the major starting marks are the development of smallpox vaccine by the English eighteenth-century physician Edward A. Jenner and the discovery of antibiotics by the nineteenth-century French scientist Louis Pasteur. During that time the current division of medical disciplines was coined, mainly based on human anatomy first described in detail by the sixteenth-century physician and scholar Vessalius. As a result most of the medical departments in hospitals around the globe are currently named after specific organ systems (such as the department of cardiology) and tissues (such as the dermatology department). Diseases were also classified according to the major affected organ or tissue. However, the increase in human lifespan during the nineteenth and twentieth centuries was accompanied by an elevated frequency of age-related complex disorders, some of which were not readily classified in terms of treatment. For example, diabetic patients are normally treated by internal medicine specialists in endocrinology; but as these patients develop the common diabetic complications, i.e. cardiovascular diseases, nephropathy, and retinopathy, other specialists have to be involved. In the lack of directed specialty in the management of complex disorders much of the burden of the follow-up of these patients usually falls upon the family physician. The only field in which the complexity of the disease is embedded within the medical infrastructure is cancer, the tremendous variability of which is addressed within oncology departments.

The major complex disorders, such as diabetes, hypertension, the various types of cancer, and the cardiovascular family of disorders, are challenging to manage not only because of the slow adaptation of the medical infrastructure to changes. These diseases are caused by multiple changes, some of which are inherited and are termed ‘susceptibility factors’, some are somatic alterations of the genetic material, and some are environmental conditions (i.e. smoking, exposure to sunlight, exposure to various chemicals, etc.). Deciphering the interplay of all these factors constitutes the heart of the challenge when investigating the causes of and designing treatment strategies for complex disorders.

How would one investigate the genetic basis of complex disorders? During recent years several approaches were used to tackle this problem, including the ‘common disease–common variant’ (CDCV) or ‘common disease–multiple rare variant’ (CDMRV) hypotheses. In general the first approach assumes that a common complex disorder could be caused by combinations of common alleles in multiple loci.^4^,^5^ The second hypothesis proposes the involvement of multiple loci in the disease phenotype but that single rare mutations in each of the many loci could lead to the disease.^5^ Disease association studies implement the CDCV hypothesis to discover new disease risk variants, and indeed multiple susceptibility factors were unearthed. Advances in sequencing technologies recently allowed assessment of the CDMRV hypothesis, though in small sample sizes so far.^6^ Although having a different logic, these two hypotheses have something in common: both assume that multiple paths could lead to the very same phenotype. Similar to the emergence of species, the emergence of complex diseases requires multiple steps and multiple factors that interplay and respond to natural selection. The large number of such factors, assuming no “seniority” of one factor over the others, renders isolating at least some of these multiple paths a major challenge.

## ANCIENT GENETIC VARIANTS AND GENETIC BACKGROUNDS PLAY A ROLE IN DISEASE SUSCEPTIBILITY

Similarities between the processes leading to the formation of new species and new diseases are amongst the first steps towards the justification of applying basic concepts of species evolution to investigate the genetic basis of complex disorders, but also vice versa.^3^ Importantly, evolutionary (Darwinian) medicine is not offered as an alternative to the old medical inquiry, but rather as a novel vantage point for biomedical phenomena.^7^

In 2005, Douglas C. Wallace pin-pointed the mitochondria and mitochondrial genetics as reflecting the very center of evolutionary medicine.^8^ Indeed, constituting a major player in cellular and organism metabolism the mitochondrion is a suitable candidate to respond to changing environments not only in the past but in modern times as well to raise the susceptibility to many complex disorders. This hypothesis received support from James Neel’s idea proposing 40 years ago the involvement of “thrifty genotypes” that were successful in ancient times during conditions of calorie restriction in the emergence of metabolic disorders today.^9^ Accordingly, a number of research groups, including our own, have demonstrated the association of ancient common mitochondrial DNA (mtDNA) genetic backgrounds with altered susceptibility to diabetes and its complications.^10^–^12^ Other complex and age-related disorders were also identified as being associated with mtDNA variation (recently reviewed).^13^ Not only correlative evidence supports this line of thinking but also experimental findings establishing the functionality of certain human mitochondrial genetic variants, thus revealing them to serve as the “radar” of natural selection.^14^,^15^ Therefore, apparently, certain positively selected functional mutations in our phylogenetic history today play a role in disease susceptibility.

The association of ancient genetic variants with disease susceptibility is not unique to the mitochondria but is common to all disease association studies, which are based on the CDCV hypothesis. The uniqueness of mitochondrial involvement in complex disorders stems mainly from the higher magnitude of mutation accumulation in the mtDNA compared to the nuclear DNA. Obviously, this fact results in increased genetic variability due to high fixation rate of mutations thus generating a large mutational repertoire to be sifted through by natural selection. Moreover, mtDNA-encoded factors are in close contact with nuclear DNA-encoded elements, especially within the oxidative phosphorylation and mitochondrial protein translation systems. This epistatic relationship, frequently termed cytonuclear interactions, is directly affected by the large difference in mutation fixation rates of the two genomes, which leads to tight co-evolution of mtDNA and nuclear DNA-encoded factors.^16^ Thus, cytonuclear interactions were implied to play a major role in adaptive and other evolutionary processes^2^,^16^–^18^ as well as in diseases.^19^ However, the rapid occurrence rate of mtDNA mutations also results in an increased repertoire of mutated mtDNAs inside the cell during the individual’s lifetime, thus further diversifying the mitochondrial genetic repertoire per cell (heteroplasmy). Hence, mitochondrial genetics is not only affected by its maternal mode of inheritance and high rate of mutation fixation during evolution but also by “intracellular” population genetics.

Heteroplasmy is a known phenomenon in mitochondrial genetics, and different levels of heteroplasmy correlate with disease severity and penetrance.^20^ Mixed populations of mtDNA molecules could be inherited from the maternal line, though its intracellular variability is thought to be bottleneck-controlled during the maternal germ-line formation,^21^,^22^ a mechanism that has recently been challenged.^23^ In contrast, heteroplasmy due to mutation accumulation during the individual’s lifetime has been supported by multiple lines of evidence, and its contribution to age-related disorders has been highlighted.^8^ Moreover, mutations may accumulate even faster in certain mitochondrial diseases in which the mtDNA replication and repair mechanisms are impaired^24^ and in various types of cancer.^25^,^26^ Both in the impaired mtDNA repair/replication diseases and in cancer the repertoire of heteroplasmic mutations is expected to be increased.^24^ This is when natural selection is engaged. We showed that *de novo* mutational combinations that became fixed in cancer tissues (head and neck squamous cell carcinoma and pancreatic cancer) strikingly tend to recapitulate ancient mtDNA mutational combinations that define established genetic backgrounds – haplogroups.^27^ Recent deep sequencing (massive parallel sequencing) of whole mtDNAs from colorectal cancer and normal adjacent tissues from 10 different individuals revealed clear differences in the repertoire of under-represented (heteroplasmic) mutations in the normal versus the disease tissue.^28^ Alternatively, a close inspection of the published heteroplasmic mutation list per individual in the last-mentioned study drew our attention to apparent notable recurrent representations of mutations that recapitulate known fixed common mtDNA variants such as those in nucleotide positions 16126, 4216 (both of which associate with mtDNA haplogroups J and T), and position 72 (which associates with mtDNA haplogroup V) (supplementary table 6 in He et al.^28^). In that case not only do the principles of evolution apply to the study of complex disorders such as cancer, but the very same mutations could play a role in both malignant and normal evolutionary processes. Although based on the assembly of multiple short (∼50 bp) sequence reads, next-generation sequencing methods provide a high resolution for the inspection of intracellular populations of molecules thus enabling the identification of relatively rare mutations which were previously invisible. Moreover it sets the basis to investigate the process of mutational fixation at the cellular and individual levels prior to their fixation in the species population. This will enable not only the assessment of the roles of natural selection and genetic drift in the mutations fixation process at the cellular level but will also pave the path towards investigating the origin of mitochondrial disease-causing mutations, many of which remain in the heteroplasmic state.

The elevated mutation (fixation) rate in cancer and certain mitochondrial diseases raises the question of the evolutionary advantage of the already high mtDNA mutation rate in healthy conditions. Above I argued that one of the pillars of the evolutionary theory is the continuous formation of genetic variability. Being the most variable coding region in the human genome, the mtDNA was thought to play a role in major evolutionary processes.^2^ The increased mutation rates in mitochondrial diseases and cancer lead me to hypothesize that the mtDNA mutation rate has a threshold beyond which the capability of the mitochondria to adapt and retain normal activities might be adversely affected. When such a putative threshold is crossed, energy metabolism is affected thus leading either to metabolic disorders, cancer, or aging.^29^,^30^ Alternatively, in cancer cells it is possible that the malignant increased mtDNA mutation rate could be part of an adaptive process thus creating novel variants in a rate high enough to allow the accumulation of a large somatic variation and response to the strong selective constraints within the lifetime of a single individual. This prediction will be testable when the cancer *de novo* mtDNA mutation data sets expand significantly as more mtDNA sequences from cancer and corresponding normal tissues are obtained.

Thus far ∼100 disease-causing mutations were described in the mitochondrial genome and, as mentioned above, the pathological phenotype of which occurs at various levels of heteroplasmy.^31^ Recently the 3243 A>G mutation causing myoclonic epilepsy and stroke-like episodes (MELAS) was found in low concentration in a notable portion of Caucasians,^32^ thus raising the possibility that these mutations are formed multiple times but only occasionally reach levels sufficient to cause a phenotype. Is the change in the level of heteroplasmy attributed to random division of the cytoplasm during cell division, i.e. intracellular genetic drift (replicative segregation), or is natural selection involved? The more next-generation sequencing technologies evolve, the more population data could be gathered – thus paving the path towards the construction of a comprehensive map of positions prone to mutagenesis and their tendency to undergo mutation fixation.

Since the repertoire of heteroplasmic mutations varies among different tissues^28^ (Buchshtav M. et al., in preparation) another dimension is added: tissue specificity. Differences in the proportion of heteroplasmic mutations could distinguish dividing tissues versus post-mitotic cells, such as blood versus muscle, respectively.^33^,^34^ Since some mitochondrial diseases exhibit tissue-specific phenotypes, such as visual loss in Leber’s Hereditary Optic Neuropathy (LHON), and since many maternally inherited diseases are caused by mtDNA mutations in a heteroplasmic state, great importance underlies the understanding of the mechanism leading to the formation of such mutations and the principles governing their occasional fixation in the mitochondrial population of different tissues. Next-generation sequencing of whole genomes such as currently generated by the 1000 Genome Project (www.1000genomes.org) and the Cancer Genome Atlas (cancergenome.nih.gov) will provide an indispensable view of the individual and tissue-specific mutational landscape and will pave the path to analysis and the generation of predictions for the functional importance and phenotype future impact of rare and common mutations. As the sequence information generated by next-generation technologies increases, our ability to assess the role of evolutionary principles in diseases becomes clearer.

## CONCLUDING REMARKS

The emerging field of evolutionary medicine faces the difficulty of implementing concepts of the long-standing theory of evolution on the rather conservative view of medicine. Such an effort was pushed forward as geneticists embarked on investigating the genetic basis of common complex disorders. As such disorders are the result of cross-talk between environmental and genetic factors, similar to major evolutionary processes of adaptation and speciation, researchers began to realize that there are commonalities in principles that govern complex diseases and evolutionary progression. One of these principles lies in the working material of evolution and natural selection – the very existence of genetic variability. In the current manuscript I pointed out the uniqueness of the mitochondria being a major matrix for genetic variability both at the population and individual levels. Technological advances in sequencing techniques (next-generation sequencing) currently allow exploring both levels of genetic variability in human mitochondria. Full assessment of intra- and interindividual sequence variability in various human populations and in various tissues in healthy and disease conditions will provide a first glance into the forces driving the formation of genetic variants. Characterizing the full extent of common and rare mutations within individuals will allow identifying the sequence alterations that have functional potential. These mutations are “sensed” by natural selection but are also excellent candidates to play a role in disease risk. Implementing the principles of evolution into understanding the genetic basis of genetic disorders constitutes the essence of evolutionary medicine and, in my unpretentious opinion, points to the future of biomedical research.

## Figures and Tables

**Figure 1.**
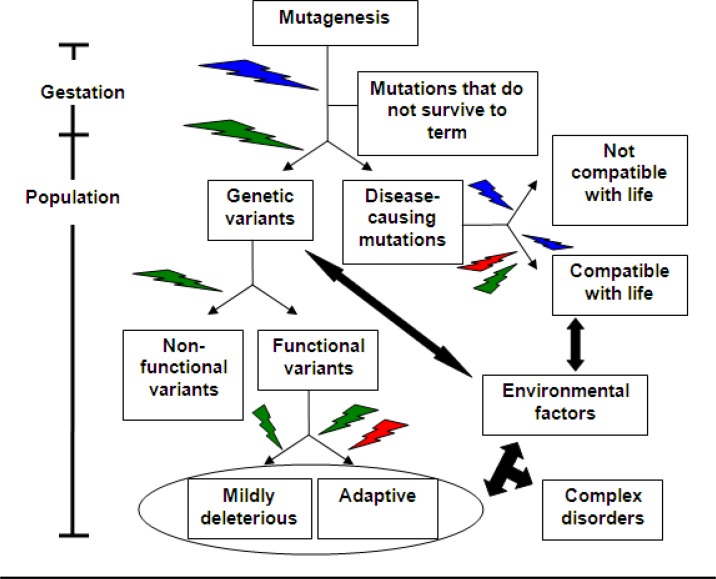
Interplay of genetic variants with evolutionary forces during embryo development and in the adult human population. Each type of mutations is subjected to different types of evolutionary forces (“lightning” arrows) at different stages: Blue = negative selection; red = positive selection; green = genetic drift.

## Abbreviations:

CDCV: common disease-common variant;
CDMRV: common disease-multiple rare variant;
LHON: Leber’s hereditary optic neuropathy;
MELAS: myoclonic epilepsy and stroke-like episodes;
mtDNA: mitochondrial DNA;
nDNA: nuclear DNA
